# Xylem vessel type and structure influence the water transport characteristics of *Panax notoginseng*

**DOI:** 10.1371/journal.pone.0281080

**Published:** 2023-03-06

**Authors:** Tianyu Xu, Zonglei Li, Sanlin Bao, Yanru Su, Zhouming Su, Shuteng Zhi, Ennan Zheng

**Affiliations:** School of Hydraulic and Electric Power, Heilongjiang University, Harbin, China; NUST: National University of Sciences and Technology, PAKISTAN

## Abstract

*Panax notoginseng* plays a very important role in medicinal and economic value. The restriction imposed by the hydraulic pathway is considered to be the main limitation on the optimal growth state of *Panax notoginseng*. The flow resistance and water transport efficiency of vessel were affected by vessel type and secondary thickening structure. The vessel structure parameters of *Panax notoginseng* were obtained by experimental anatomy, and the flow resistance characteristics were analyzed by numerical simulation. The results showed that the xylem vessels had annular thickening and pit thickening walls. The flow resistance coefficient (*ξ*) of the pitted thickening vessel was significantly lower than that of annular thickening vessel in four cross-sectional types. The *ξ* of the circular cross-sectional vessel was the largest, followed by the hexagon, pentagon cross-sectional vessel and the lowest was the quadrilateral cross-sectional vessel, and the structure coefficient (*S*) was just the opposite. The *ξ* of the vessel model was positively correlated with the annular height, pitted width and pitted height, and negatively correlated with the annular inscribed circle diameter, annular width, annular spacing, pitted inscribed circle diameter and pitted spacing. Among them, annular (pitted) height and the annular (pitted) inscribed circle diameter had a great influence on the *ξ*. The increasing and decreasing trend of the *S* and *ξ* were opposite in the change of annular (pitted) inscribed circle diameter, and consistent in the change of in other structural parameters, indicating that the secondary wall thickening structure limited the inner diameter of the vessel to maintain a balance between flow resistance and transport efficiency.

## Introduction

*Panax notoginseng* is an herbaceous plant in the Araliaceae family. It is the main cash crop planted widely in seasonal arid regions [1], which has high medicinal value, and the economic income of *Panax notoginseng* cultivation is about 500 thousand RMB/ha [2]. The growth of *Panax notoginseng* has strict requirements on temperature and humidity conditions [3]. The limitation of the hydraulic pathway is considered to be the major constraint on the growth of *Panax notoginseng* [4, 5]. Water transport from the roots to the leaves depends on the xylem, and the vessel is the main water transport channel of xylem [6]. The relationship between water transport efficiency and vessel micromorphology can be considered as an important indicator for identifying plant growth [7, 8]. The structure of the vessel inner wall is dependent on the local deposition of wall material during the development of the secondary wall, which will show various patterns on the inner wall, such as annular, helical, reticulated and pitted thickening [9]. Chen Qi et al. [10] used the SST k-ε model to analyze the flow resistance of annular and helical thickening structures of the inner wall, and the structural flow resistance of the helical thickening vessel was less than that of annular thickening, indicating the secondary wall thickening of xylem directly affects water conductivity by the vessel [11].

The size of the xylem vessel is between micrometers and millimeters and often structurally complex [12, 13]. The internal flow of vessel structure is more difficult to measure by plant physiology. Ulyana S. et al. [14] used contrast-enhanced magnetic resonance imaging (MRI) and laser scanning microscopy to reveal the structural and dimensional characteristics of the maize stem vascular system. Visualization and quantification of spatial xylem organization of the angiosperm species *Fraxinus excelsior L*. on the microscopic level by X-ray micro-computed tomography. It was possible to determine morphological characteristics of the cellular axial tissue three-dimensionally [15]. Many scholars have studied the flow mechanism of the xylem vessel microstructure using computational fluid dynamics (CFD). Qu W et al. [16] used the lattice Boltzmann method (LBM) and curved boundary treatment to analyze the relationship between the pit structure and its hydraulic characteristics. The results showed that the flow rate of each pore is the result of the combination of pore area and pore radial position. Daniel et al. [17] used numerical simulations to analyze the xylem water ascent of African mahogany (*Khaya grandifoliola*) cultivated under different irrigation regimes. It was found that the non-irrigated vessels had a higher number of pits in the secondary wall thickening when compared to the irrigated treatments. Xu et al. [18] analyzed the water transport characteristics of *Jatropha curcas* xylem vessels with perforation plate and secondary wall thickening that the smooth vessel resistance accounted for the largest proportion of total resistance.

A number of experimental and simulation data showed that the vessel structure should play a prominent role in determining the efficiency of water supply to the xylem [19, 20]. Most studies have focused on water transport in circular cross-section vessel [18, 21]. The cross-sectional type affects the vessel structure and changes the resistance in water transport. In fact, xylem vessels exist in a variety of cross-sectional forms that leads to variability among water transport efficiencies.

In this paper, anatomical experiments were conducted to observe that the xylem vessels of *Panax notoginseng* have quadrilateral, pentagonal, hexagonal and circular cross-sections. In order to further explore the relationship between the vessel structure and water transport efficiency, the scanning electron microscopy (SEM) were used to obtain inner wall thickening parameters, combined with numerical simulations to establish the mathematical model for calculating the flow resistance. It can: (1) obtain the distribution law of the flow field inside the vessel and the influence of the parameters on the flow characteristics; (2) evaluate the relationship among the cross-sectional type, secondary wall thickening and the flow resistance coefficient; (3) analyze the effect of cross-sectional type and vessel structural parameters on structure coefficient. The results will provide a reference for the water transport efficiency of *Panax notoginseng* and promote a deeper understanding of water transport regularity in xylem.

## Materials and methods

### Preparation of sample and anatomical methods

Experimental materials: The *Panax notoginseng* was sampled at Kunming University of Science and Technology in Yunnan Province, which is located 24°84′46″N and 102°86′38″E at an elevation of 1860 m above sea level. It belongs to the northern subtropical monsoon climate zone. The soil of the experimental site was clay and slightly acidic red loam, and the field water holding capacity was 30.34%. There were 25 ridges in the greenhouse, with a width of 150cm, a length of 420cm and a row spacing of 10cm×12cm (width×length). About 525 *Panax notoginseng* plants were planted in one ridge. The 8–10 samples of the stems were randomly collected, the height of the stem sampling level was between 10-20cm, embedded in paraffin and sliced within 3–4 days and analyzed by scanning electron microscopy (SEM) [18].

Paraffin embedding method: The stems of the sample were cut into 2cm long pieces with a hand knife, fixed in formaldehyde for half an hour and then dehydrated. The samples were put into 75%, 90%, 95% and 100% ethanol solutions (20 minutes) to dislodge the moisture. The dehydrated samples were put into the xylene-absolute ethanol mixture for 1 hour, and then put into the xylene solution for 2 hours. The samples dipped in wax for 2 hours before embedding treatment. The embedded samples were sliced (cross-section and axial section) by the Leica 2000R slide-away microtome, the thickness of the sections was generally 10 to 20 μm, and the sliced samples were placed in xylene for deparaffinization [22].

**Fig 1:**
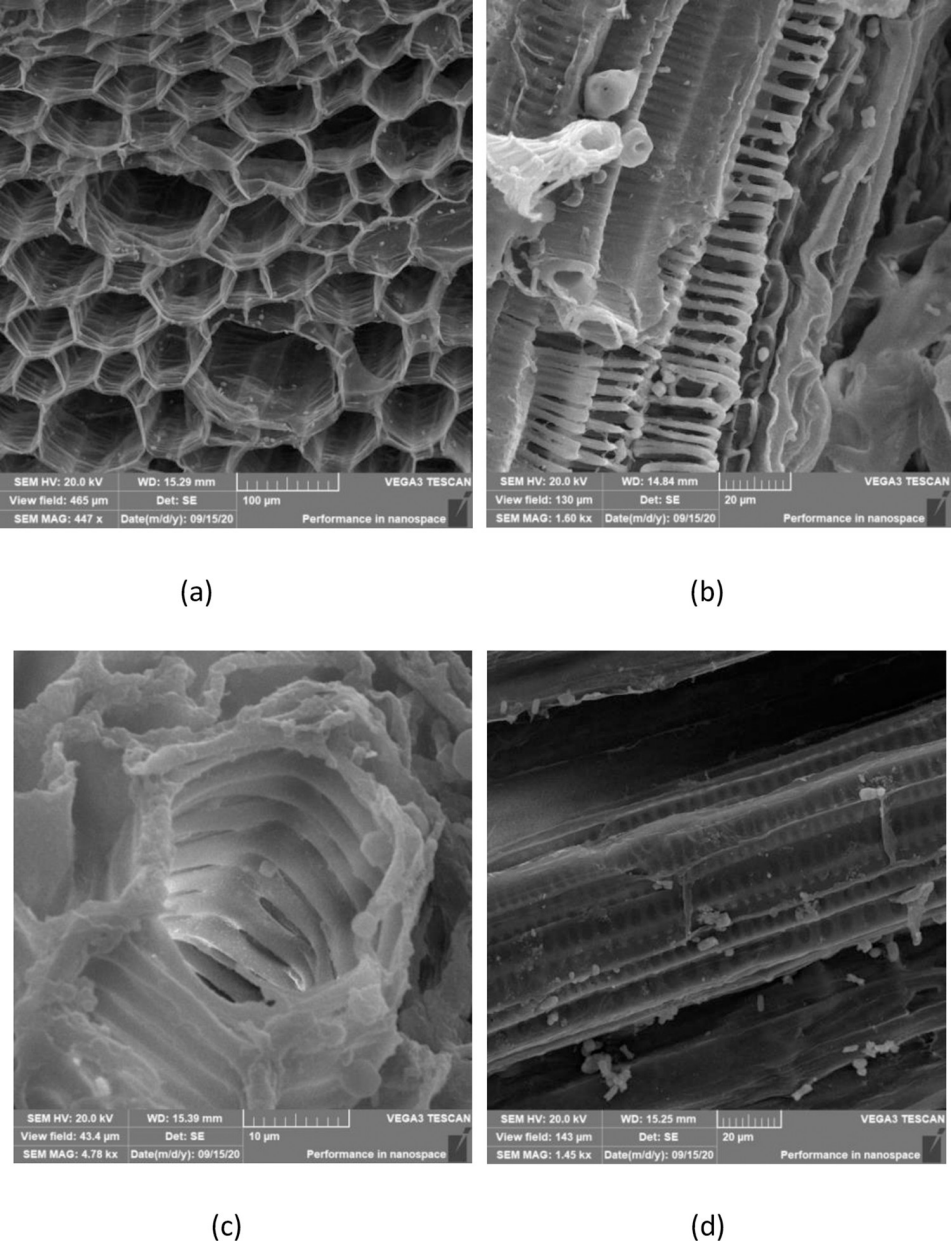
Schematic diagram of annular thickening and pitted thickening structure of the *Panax notoginseng*. (a)The cross-section of annular thickening, (b)The axial-section of annular thickening, (c)The cross-section of pitted thickening, (d)The axial-section of pitted thickening.

Observation method: The samples were fixed on the observation platform by conductive glue after metal spraying (vacuum ion sputtering instrument), and the observation space was vacuum sealed for tungsten filament scanning electron microscope (SEM) observation. The cross section of the sample was used to observe the cross-sectional type, inscribed circle diameter and thickening height (Fig 1A and 1C). The axial section of the sample was used to observe the thickening width and spacing (Fig 1B and 1D).

### Model construction of xylem vessel

The detailed structural parameters of the *Panax notoginseng* xylem were shown in the scanning electron microscopy images. The flow resistance characteristics of the vessel were analyzed by the computational fluid dynamics method [11, 18]. Based on the microscopic images of the cross-section and axial-section, the structural parameters of the annular thickening and pitted thickening vessel were measured. The measurements were taken for each character listed in Table 1.

**Table 1:** Structural parameters of the *Panax notoginseng*.

Structural parameters	Annular thickening	Structural parameters	Pitted thickening
Cross section	Hexagon/Quadrilateral/Pentagon/Circular	Cross section	Hexagon/Quadrilateral/Pentagon/Circular
vessel length	300μm	vessel length	300μm
inscribed circle diameter	20+15μm	inscribed circle diameter	20±3μm
annular height	2.4±0.2μm	Pitted length	8±2μm
annular width	2.4±0.25μm	Pitted width	4±0.5μm
annular spacing	2.4±0.2μm	pitted spacing	2±0.4μm
		Pitted height	1.2+0.4μm

**Fig 2:**
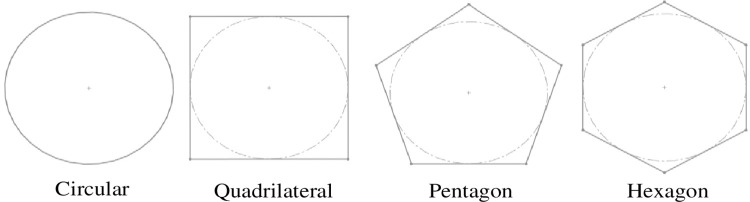
The types of vessel cross-sections.

**Fig 3:**
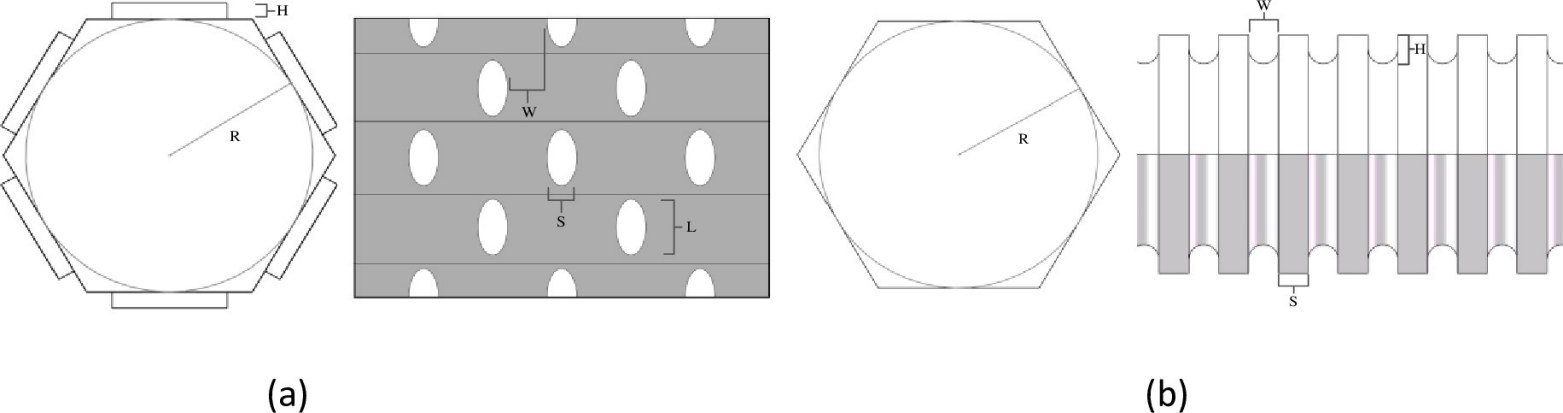
Structural parameters of the *Panax notoginseng*. (a)pitted thickening structure, (b)annular thickening structure.

The types of vessel cross-sections were shown in Fig 2. The terms of the annular thickening and pitted thickening vessel were shown in Fig 3, which *R* was inscribed circle diameter, *W* was width, *S* was spacing, *H* was height, *L* was length.

**Fig 4:**
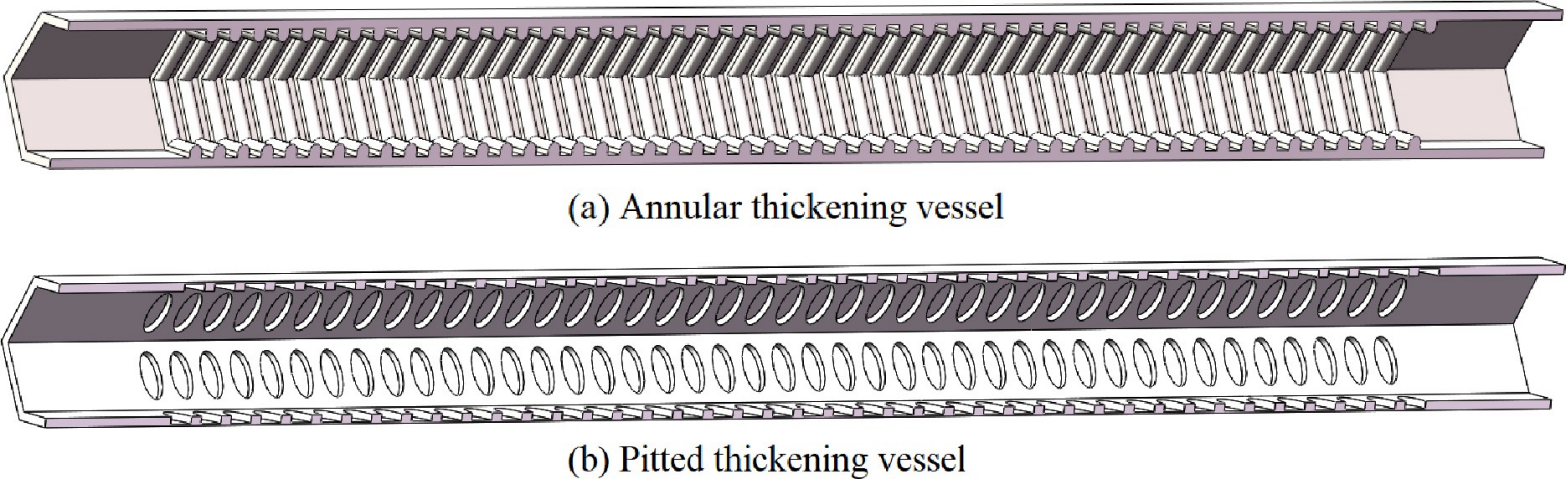
Schematic diagram of *Panax notoginseng* model. (A) Annular thickening vessel. (b) Pitted thickening vessel.

The actual structural parameters of the xylem were obtained from the *Panax notoginseng* samples (Table 1), and the computational domain model of the annular thickening and pitted thickening vessel was established based in SolidWorks. The hexagon vessels of *Panax notoginseng* were shown in Fig 4.

**Fig 5:**
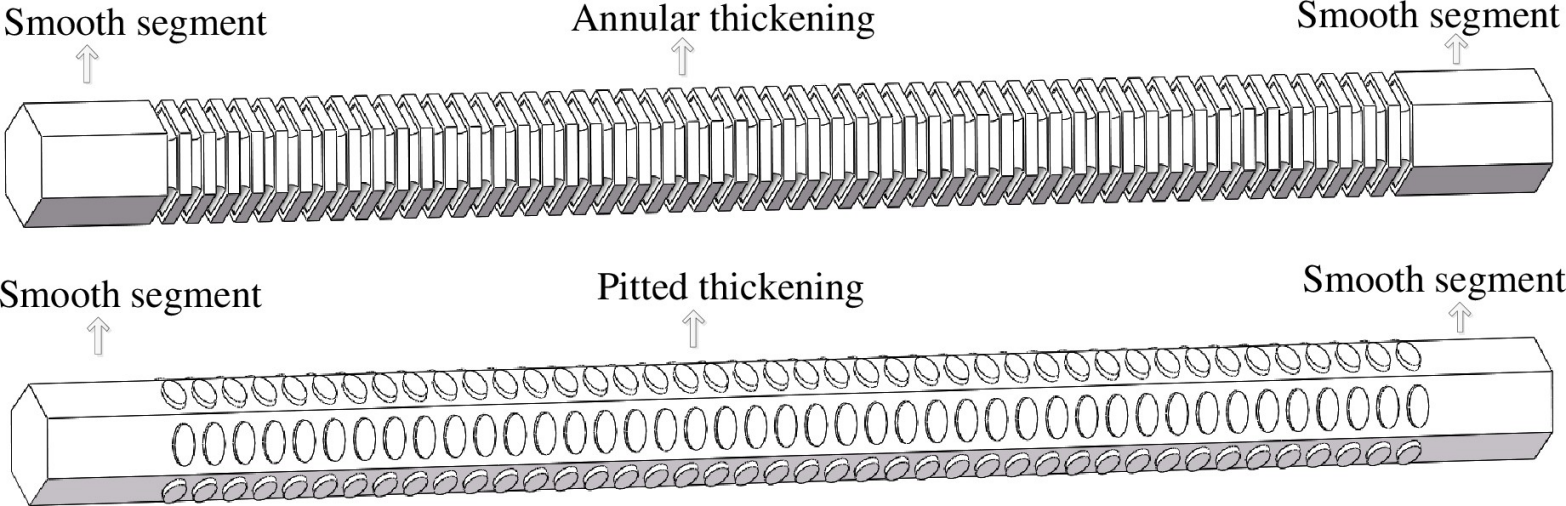
Fluid domain calculation model of vessel.

The computational domain models contained a flow area with a secondary wall thickening pattern of 250 μm in length. To avoid effects at the entrance and exit, an extended smooth segment with length 25 μm was added at both ends of the vessel (Fig 5).

**Fig 6:**
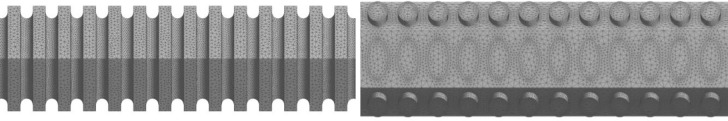
The mesh of the vessels.

The boundary conditions were that the pressure was zero at the model outlet, and the flow velocity was 0.3 mm/s at the model inlet. The irregularity of the vessel structure was generated by tetrahedral and hexahedral unstructured meshes. The maximum and minimum of the unit size were 4.4×10^-6^m and 4.4×10^-8^m, respectively. In this part, the scale of the mesh was based on the prediction accuracy of the inlet/outlet pressure drop, and the mesh size independence test was performed (Table 2). The pressure drop difference between the standard mesh and the fine mesh was 0.22%. The mesh number has no effect on the calculation results, so the standard mesh number was used to analyze flow resistance characteristics, and the total number of meshes in the model was approximately 728680 (Fig 6). The PowerCube-S01 with a high-performance computing system was used for the simulation.

**Table 2:** Mesh size independence test.

	Mesh number	Pressure drop difference
Coarser	157824	--
Coarse	360426	1.16%
Standard	728680	0.49%
Fine	1507459	0.22%

### Calculation method of flow resistance coefficient

**Fig 7:**
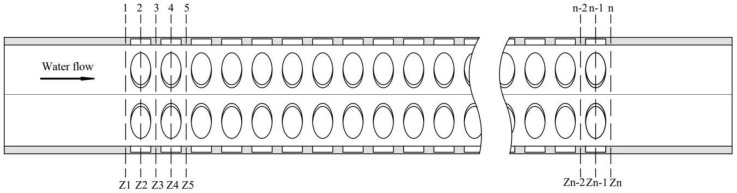
Schematic of water flow in the pitted thickening vessel.

The hexagonal xylem vessel model (Fig 7) can analyze the flow characteristics by the energy conservation law (Bernoulli equation). The flow between arbitrary sections satisfies the Bernoulli equation, which was written in sections from the inlet to the exit sections *Z*_*1*_, *Z*_*2*_, *···*, *Z*_*n*_ as:

$$\begin{array}{c} \frac{P_{1}}{\rho g}+\frac{V_{1}^{2}}{2g}+z_{1}=\frac{P_{2}}{\rho g}+\frac{V_{2}^{2}}{2g}+z_{2}+\xi_{1}\frac{V_{2}^{2}}{2g}+\lambda \frac{l_{1}V_{2}^{2}}{8Dg}\\ \frac{P_{2}}{\rho g}+\frac{V_{2}^{2}}{2g}+z_{1}=\frac{P_{3}}{\rho g}+\frac{V_{3}^{2}}{2g}+z_{3}+\xi_{2}\frac{v_{3}^{2}}{2g}+\lambda \frac{l_{2}V_{3}^{2}}{8Dg}\\ \cdot \cdot \cdot \cdot \cdot \cdot \\ \cdot \cdot \cdot \cdot \cdot \cdot \\ \frac{P_{n-1}}{\rho g}+\frac{V_{n-1}^{2}}{2g}+z_{n-1}=\frac{P_{n}}{\rho g}+\frac{V_{n}^{2}}{2g}+z_{n}+\xi_{n-1}\frac{V_{n}^{2}}{2g}+\lambda \frac{l_{n-1}V_{n}^{2}}{8Dg} \end{array}$$ (1)

Where *P*_*n*_ and *V*_*n*_ were the average pressure and flow velocity at section n, *ρ* was fluid density, *g* was the acceleration of gravity, *Z*_*n*_ was the position head of water at the section, *ξ*_*n−1*_ was the local loss coefficient of section *n-1* to section *n*, *λ* was friction factor of head loss, *l*_n−1_ was the length between two adjacent sections. *D* was the hydraulic radius of the xylem vessel, the expression of *D* was:

$$D=\frac{A}{\chi }$$ (2)

Add the two sides of the equations of Eq (1) in order:

$$\frac{P_{1}-P_{n}}{\rho g}=z_{n}-z_{1}+\xi_{1}\frac{V_{2}^{2}}{2g}+\xi_{2}\frac{V_{3}^{2}}{2g}+\cdots +\xi_{n-1}\frac{V_{n}^{2}}{2g}+\lambda \frac{LV_{n}^{2}}{8Dg}$$ (3)

Where *l*_*1*_*+l*_*2*_*+l*_*3*_*+···+l*_*n-1*_ *= L*, *L* was the total length of the xylem vessel.

Known by the continuity equation:

$$V_{1}A_{1}=V_{2}A_{2}=V_{3}A_{3}=\cdots =V_{n}A_{n}$$ (4)

In Eq (4), *A*_*i*_*(i = 1*,*2*…,*n)* was the flow area at the corresponding section, Substituting Eq (4) into Eq (3) give:

$$\frac{\Delta P}{\rho g}=L+[\lambda {\left(\frac{A_{1}}{A_{n}}\right)}^{2}\frac{L}{4D}+\sum_{i=1}^{n-1}\xi_{i}{\left(\frac{A_{1}}{A_{i+1}}\right)}^{2}]\frac{V_{1}{}^{2}}{2g}$$ (5)

Where

$$\xi =[\lambda {\left(\frac{A_{1}}{A_{n}}\right)}^{2}\frac{L}{4D}+\sum_{i=1}^{n-1}{\left(\frac{A_{1}}{A_{i+1}}\right)}^{2}\xi_{i}]$$ (6)

Eq (6) was simplified to:

$$\frac{\Delta P}{\rho g}=L+\xi \frac{V_{1}^{2}}{2g}$$ (7)

Expressed as:

$$\xi =\frac{2}{V_{1}^{2}}(\frac{\Delta P}{\rho }‐Lg)$$ (8A)

Expressed by flow rate:

$$\xi =\frac{24R^{4}}{q^{2}}(\frac{\Delta P}{\rho }-Lg)$$ (8B)

In Eqs (8A, 8B), was the flow resistance coefficient of hexagonal xylem vessel, *q* was the average flow rate.

For pentagon, quadrilateral and circular xylem vessel model, the expressions were:

$$\xi =\frac{50R^{4}{\left(\tan36^{\circ }\right)}^{2}}{q^{2}}(\frac{\Delta P}{\rho }-Lg)$$ (8C)

$$\xi =\frac{32R^{4}}{q^{2}}(\frac{\Delta P}{\rho }-Lg)$$ (8D)

$$\xi =\frac{2\pi^{2}R^{4}}{q^{2}}(\frac{\Delta P}{\rho }-Lg)$$ (8E)

## Results

### Velocity and pressure distribution of fluid in the vessel

**Fig 8:**
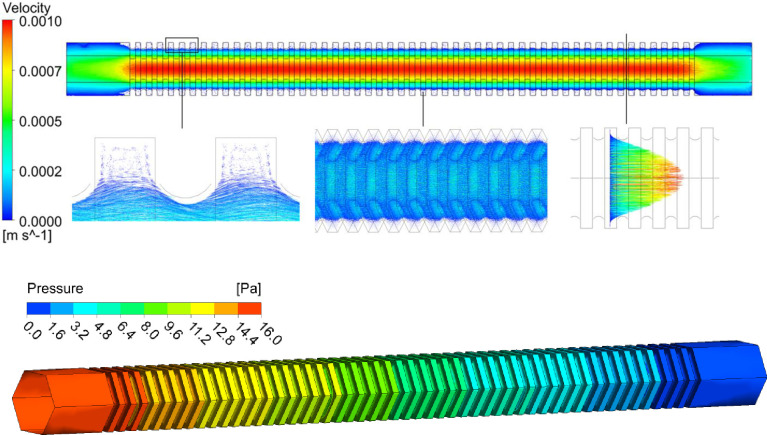
Velocity and pressure distribution of fluid in the annular thickening vessel.

**Fig 9:**
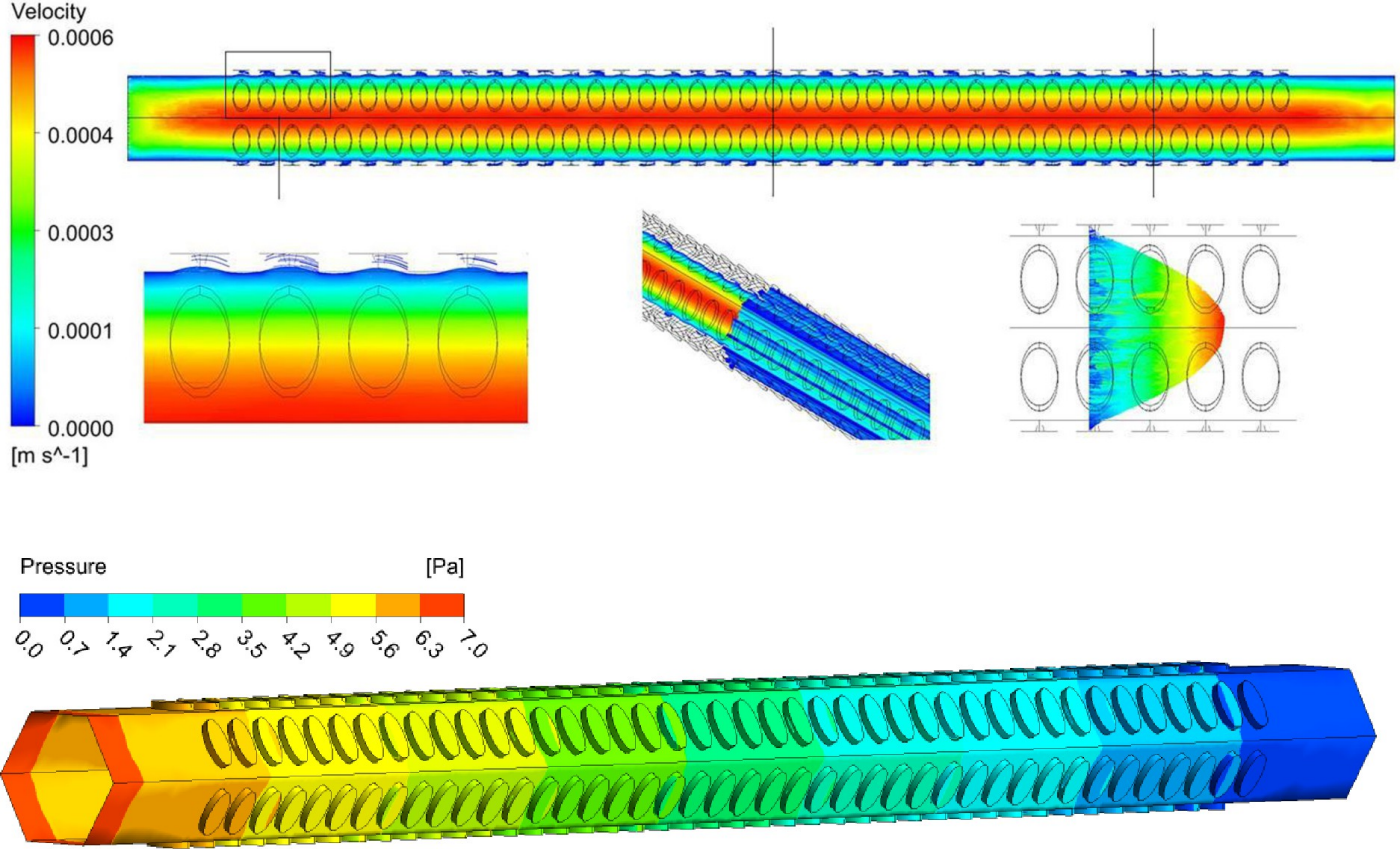
Velocity and pressure distribution of fluid in the pitted thickening vessel.

The flow and pressure profiles of the annular thickening vessel and the pitted thickening vessel model were analyzed. The velocity and pressure at all points in the annular thickening vessel and pitted thickening vessel were not the absolute flow velocities and pressures in the *Panax notoginseng* xylem. However, the comparability velocities and pressures within different regions were valid. In the annular thickening vessel and pitted thickening vessel, the flow velocity was distributed in a gradient along the radial direction of the vessel, with the maximum velocity in the central axis and gradually decreasing to both sides, showing an obvious laminar flow state (Figs 8 and 9). There was a low-speed vortex area in the section of the annular thickening, which reduced the equivalent inner diameter and affected the maximum velocity in the axial region. There was no low-speed vortex area in the section of the pitted thickening, which had little effect on the maximum flow velocity in the axial center area. Therefore, the pressure gradient on both sides of the annular thickening structure was more obvious than that of the pitted thickening structure. It can be seen from Figs 8 and 9 that the overall pressure was distributed in a gradient along the axial direction of the vessel, with the maximum pressure at the front end of the vessel and gradually decreasing towards the back end. The overall pressure of the annular thickening structure was larger than that of the pitted thickening structure.

### Flow resistance characteristics of cross-sectional types

Via the numerical simulation, the total pressure (*Δp*) and the average flow rate (*q*) were obtained. The *ξ* of the xylem vessel was calculated according to the Formulas (8B,8C,8D and 8E). The water transport of *Panax notoginseng* relied on xylem vessels, and cross-sectional types of xylem vessels had a significant impact on water transport.

It can be seen from Table 3 that the *Δp*, *q* and *ξ* of the polygonal vessel with the same inscribed circle were different. In annular thickening and pitted thickening vessels, The circular vessels had the largest *Δp* and *ξ*, followed by hexagon, pentagon and quadrilateral vessels, and the *q* was the opposite. The *Δp* and *ξ* in an annular thickening vessel of the same cross-sectional type was greater than that the pitted thickening vessel. In annular thickening vessels, the *Δp* and *ξ* of the circular vessels were 6.83% and 21.26% higher than that of quadrilateral vessels, and the *q* of the circular vessels was 25.83% less than that of quadrilateral vessels. In pitted thickening vessels, the *Δp* and *ξ* of the circular vessels were 4.65% and 22.28% higher than that of quadrilateral vessels, and the *q* of the circular vessels was 25.83% less than that of quadrilateral vessels.

**Table 3:** Flow resistance coefficient of xylem vessel in the stems.

Models	Annular thickening			Pitted thickening		
	Δp/Pa	q/(m^3^·s^–1^)	*ξ*	Δp/Pa	q/(m^3^·s^–1^)	*ξ*
Quadrilateral	14.34	1.20×10^−13^	2.54×10^5^	6.24	1.20×10^−13^	7.36×10^4^
Pentagon	14.88	1.09×10^−13^	2.66×10^5^	6.43	1.09×10^−13^	7.78×10^4^
Hexagon	15.19	1.04×10^−13^	2.73×10^5^	6.53	1.04×10^−13^	8.00×10^4^
Circular	15.32	8.90×10^−14^	3.08×10^5^	6.56	8.90×10^−14^	9.00×10^4^

### The vessel structure parameters on flow resistance coefficient

#### Annular thickening vessel parameters on flow resistance coefficient

**Fig 10:**
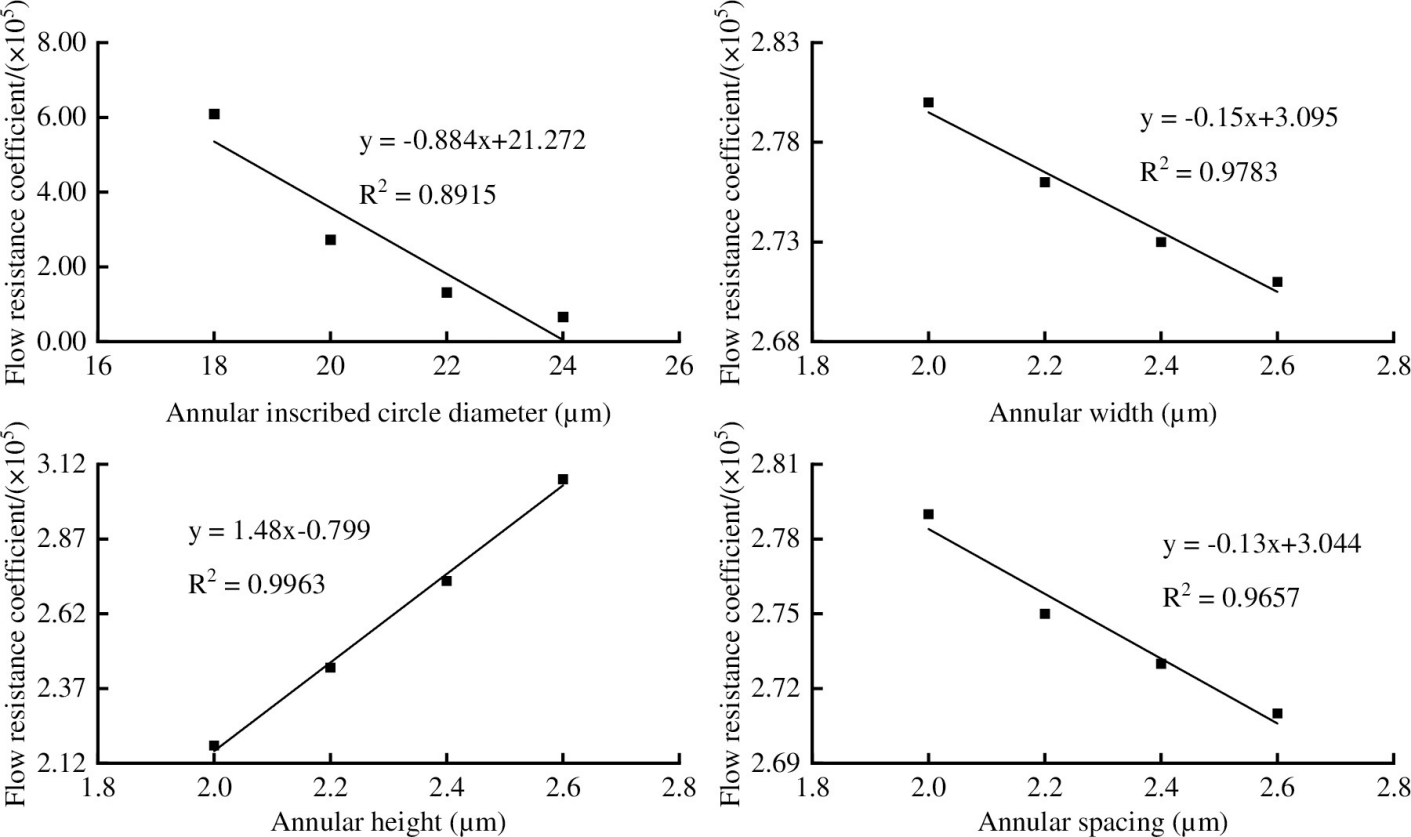
The annular thickening vessel parameters on flow resistance coefficient: (a) annular inscribed circle diameter, (b) annular width, (c) annular height, (d) annular spacing.

The *Δp*, *q* and *ξ* were affected by the annular inscribed circle diameter, annular width, annular height, and annular spacing. There were close linear relationship between the *ξ* with annular inscribed circle diameter, annular width, annular height, and annular spacing (Fig 10), the determination coefficient (R^2^) were 0.8915, 0.9783, 0.9963 and 0.9657, indicating that the equations had good fitting. It can be seen in Fig 10 that the *ξ* decreased with the increase of annular inscribed circle diameter, annular width and annular spacing, and increased with the increase of annular height. When the annular inscribed circle diameter changed from 18 to 24 μm, the *Δp* changed from 20.86 to 9.13 Pa, the *q* changed from 8.40×10^−14^ to 1.49×10^−13^ m^3^/s, and the *ξ* changed from 6.09×10^5^ to 0.67×10^5^, which decreased by 89.00%. When the annular width changed from 2.0 to 2.6 μm, the *Δp* changed from 15.49 to 15.09 Pa, the *q* remained constant, and the *ξ* changed from 2.80×10^5^ to 2.71×10^5^, which decreased by 3.21%. When the annular height changed from 2.0 to 2.6 μm, the *Δp* changed from 12.71 to 16.71 Pa, the *q* remained constant, and the *ξ* changed from 2.18×10^5^ to 3.07×10^5^, which increased by 40.83%. When the annular spacing changed from 2.0 to 2.6 μm, the *Δp* changed from 15.47 to 15.10 Pa, the *q* remained constant, and the *ξ* changed from 2.79×10^5^ to 2.71×10^5^, which decreased by 2.87%.

#### Pitted thickening vessel parameters on flow resistance coefficient

**Fig 11:**
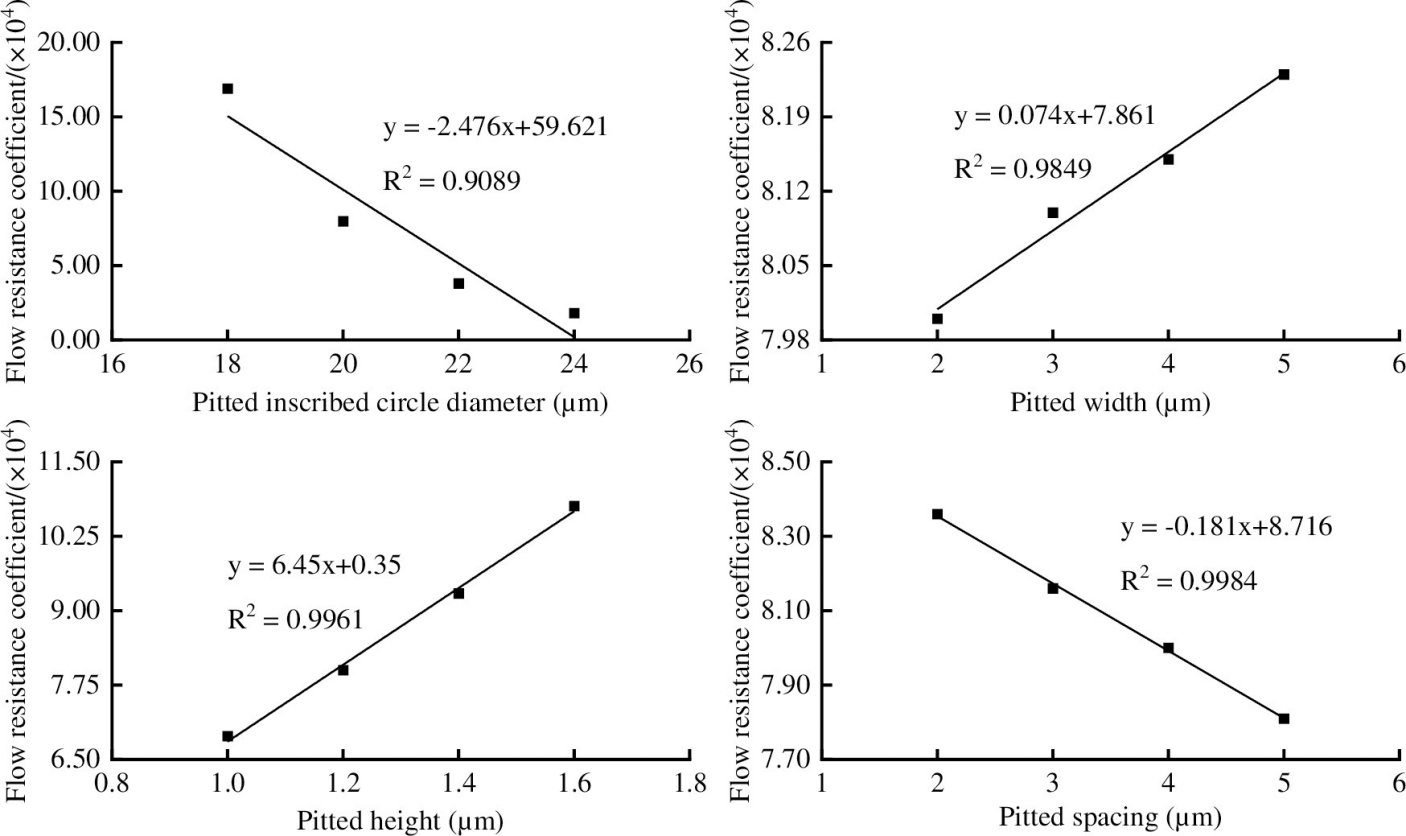
The pitted thickening vessel parameters on flow resistance coefficient: (a) pitted inscribed circle diameter, (b) pitted width, (c) pitted height, (d) pitted spacing.

The *Δp*, *q* and *ξ* were affected by the pitted inscribed circle diameter, pitted width, pitted height, and pitted spacing. There were close linear relationship between the *ξ* with pitted inscribed circle diameter, pitted width, pitted height, and pitted spacing (Fig 11), the determination coefficient (R^2^) were 0.9089, 0.9849, 0.9961 and 0.9984, indicating that the equations had good fitting. It can be seen in Fig 11 that the *ξ* decreased with the increase of pitted inscribed circle diameter and pitted spacing, and increased with the increase of pitted width and pitted height. When the pitted inscribed circle diameter changed from 18 to 24 μm, the *Δp* changed from 7.92 to 4.62 Pa, the *q* changed from 8.40×10^−14^ to 1.49×10^−13^ m^3^/s, and the *ξ* changed from 1.69×10^5^ to 0.18×10^5^, which decreased by 89.35%. When the pitted width changed from 2.0 to 5.0 μm, the *Δp* changed from 6.69 to 6.45 Pa, the *q* remained constant, and the *ξ* changed from 8.36×10^4^ to 7.81×10^4^, which decreased by 6.58%. When the pitted height changed from 1.0 to 1.6 μm, the *Δp* changed from 6.29 to 7.04 Pa, the *q* changed from 1.08×10^−13^ to 9.56×10^−14^ m^3^/s, and the *ξ* changed from 6.89×10^4^ to 1.08×10^5^, which increased by 56.75%. When the pitted spacing changed from 2.0 to 5.0 μm, the *Δp* changed from 6.53 to 6.63 Pa, the *q* remained constant, and the *ξ* changed from 8.00×10^4^ to 8.23×10^4^, which increased by 2.79%.

### The cross-sectional types and vessel structural parameters on structure coefficient

To study the water transport efficiency of the vessel on the structure coefficient, the parameters of annular thickening and pitted thickening were analyzed. Here, the structure coefficient was expressed as the percentage of thickening structural resistance in the total resistance.

$$S=\frac{\xi -\xi_{s}}{\xi }$$ (9)

where *S* was the structure coefficient, *ξ* was the flow resistance coefficient of secondary wall thickening vessel, *ξ*_*s*_ was the flow resistance coefficient of smooth vessel.

The influence of cross-sectional types on *ξ* and *ξ*_*s*_ was shown in Table 4. The structure coefficient of the *Panax notoginseng* vessel was calculated by Eq (9). The results showed that the quadrilateral vessel had the largest structure coefficient, followed by pentagon vessel, hexagon vessel and circular vessel in annular thickening and pitted thickening vessel. The *S* of the annular thickening vessel with the same cross-section was higher than that of the pitted thickening vessel.

**Table 4:** Structure coefficient of cross-sectional types.

Models	Annular thickening			Pitted thickening		
	*ξ*	*ξ*_*s*_	S	*ξ*	*ξ*_*s*_	S
Quadrilateral	2.54×10^5^	3.76×10^4^	0.852	7.36×10^4^	1.66×10^4^	0.774
Pentagon	2.66×10^5^	4.83×10^4^	0.818	7.78×10^4^	2.52×10^4^	0.676
Hexagon	2.73×10^5^	6.04×10^4^	0.779	8.00×10^4^	3.27×10^4^	0.591
Circular	3.08×10^5^	7.37×10^4^	0.761	9.00×10^4^	4.36×10^4^	0.516

**Fig 12:**
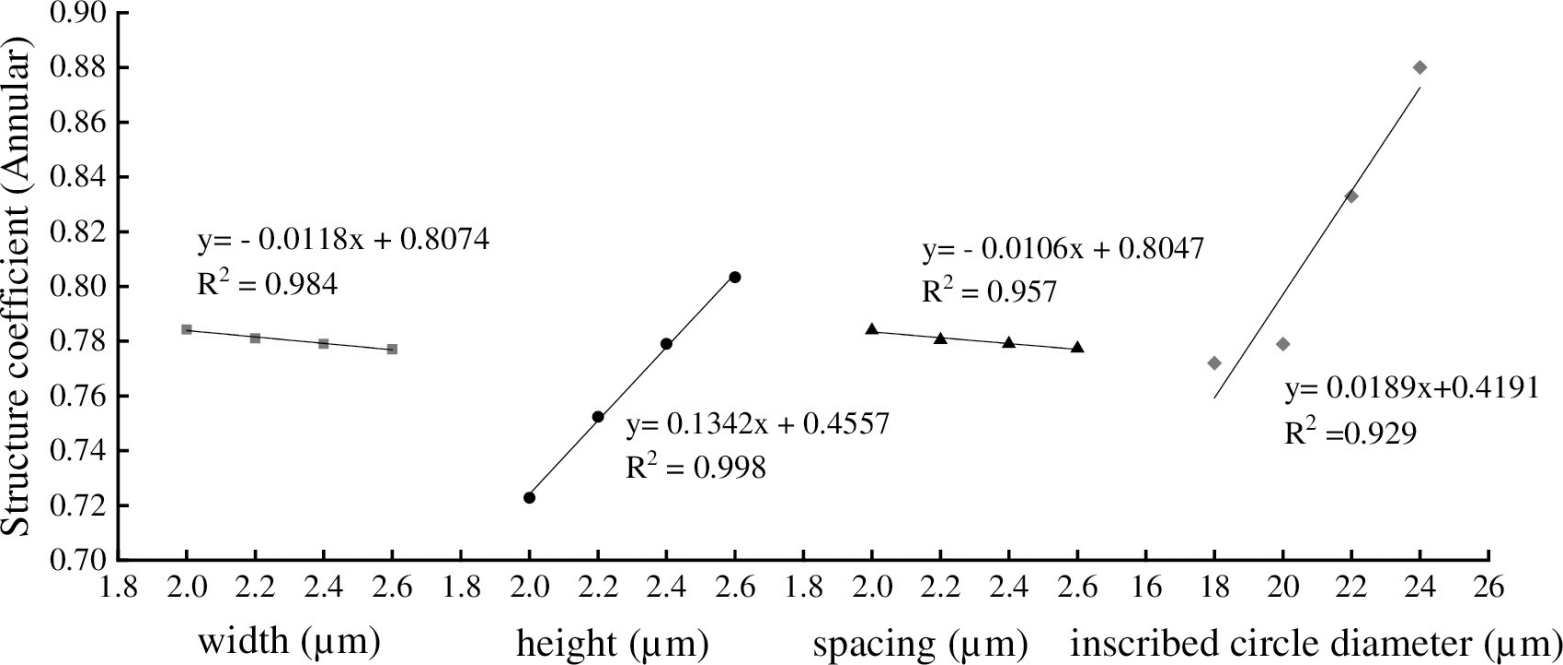
The annular thickening vessel parameters on structure coefficient.

**Fig 13:**
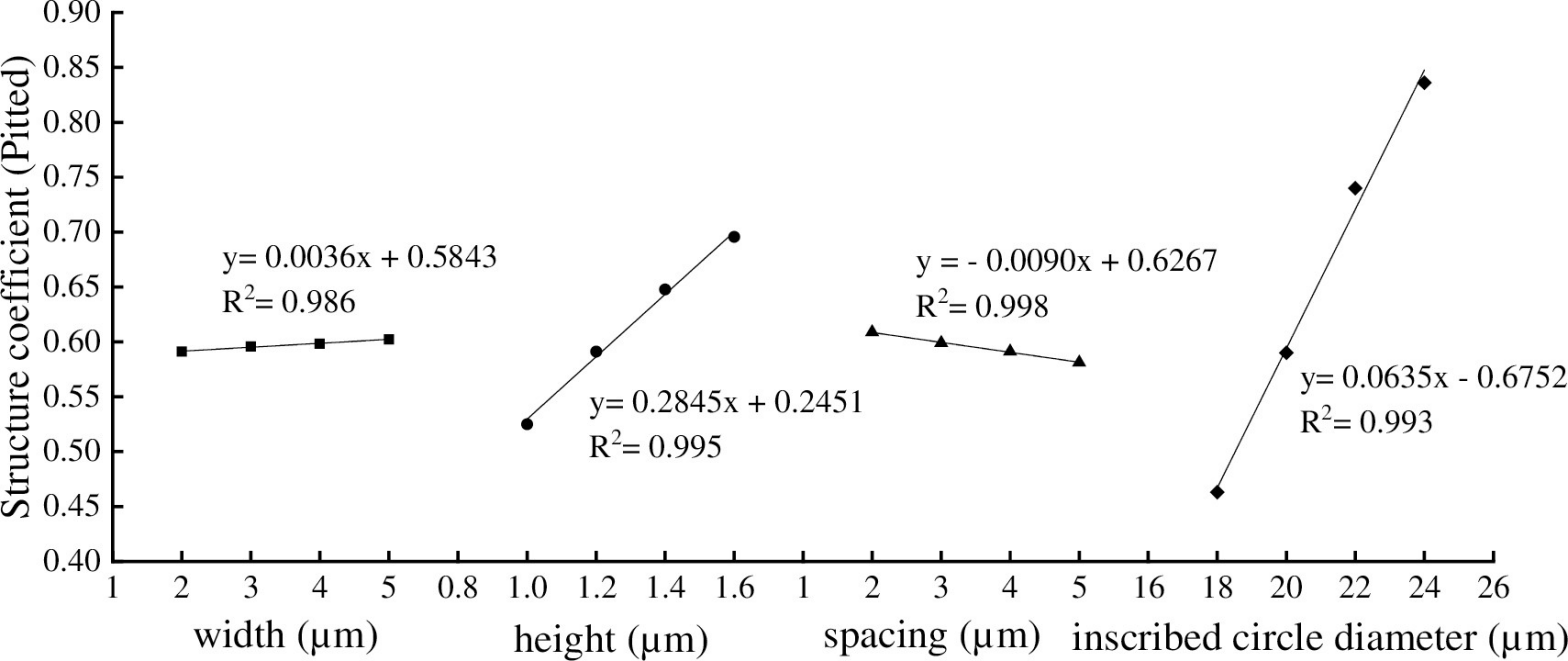
The pitted thickening vessel parameters on structure coefficient.

It can be seen from the Figs 12 and 13 that the *S* of hexagon vessel was affected by the inscribed circle diameter, width, height, and spacing of annular thickening and pitted thickening structure. There were close linear relationship between the *S* with width, height, spacing and inscribed circle diameter, the determination coefficient (R^2^) were 0.984, 0.998 0.957 and 0.929 in annular thickening and 0.986, 0.995, 0.998, 0.993 in pitted thickening, indicating that the equations had good fitting. In annular thickening vessel (Fig 12), when the annular width changed from 2.0 to 2.6 μm, the *S* decreased by 0.89%. When the annular height changed from 2.0 to 2.6 μm, the *S* increased by 11.07%. When the annular spacing changed from 2.0 to 2.6 μm, the *S* decreased by 0.89%. When the annular inscribed circle diameter changed from 18 to 24 μm, the *S* increased by 13.99%. In pitted thickening vessel (Fig 13), when the pitted width changed from 2.0 to 5.0 μm, the *S* increased by 1.86%. When the pitted height changed from 1.0 to 1.6 μm, the *S* increased by 32.38%. When the pitted spacing changed from 2.0 to 5.0 μm, the *S* decreased by 4.60%. When the pitted inscribed circle diameter changed from 18 to 24 μm, the *S* increased by 80.56%.

## Discussion

The experimental anatomy combined with the numerical simulation method will be a useful one for analyzing the flow through the vessel cross-section. The research on the vessel section mainly focused on the circular structure [10, 21]. Combined with anatomical observations, the cross-section of the vessel of *Panax notoginseng* was in many types, thus the vessel structure was complicated [20]. When the diameter of the inscribed circle of the polygonal section was the same, the flow resistance coefficient of the vessel with more polygonal structure sides was higher. The main reason was that the cross-sectional structure with more sides was closer to a circle, the inner diameter of the vessel was smaller, and the pressure drop increased, resulting in an increase of flow resistance [11]. The results showed that the flow resistance coefficient of the pitted thickening vessel was significantly lower than that of an annular thickening vessel in four cross-sectional types. The reason was that the pit structure increased the equivalent inner diameter of the vessel and reduced the flow resistance [22]. Xu et al. [18] found that the pit structure had radial water transport in the xylem vessels of *Jatropha curcas*. However, in the study of *Panax notoginseng*, it was not found that the pit structure can carry out radial water transport, which also showed that there were differences in the pit structure in plants, resulting in differences in water transport efficiency [23, 24].

It can be seen in Figs 10 and 11 that the influence of annular (pitted) height and inner diameter on the flow resistance coefficient was larger than that of annular (pitted) width and annular (pitted) spacing in hexagon vessel. The reason that the change of the equivalent inner diameter caused by the secondary wall thickening structure in the radial direction was greater than that of the axial structural resistance. Similar conclusions were obtained in the analysis of circular vessel by Chen Qi et al. and Xu et al. [10, 18]. In this paper, the structure coefficient (S) was used to analyze the change in transport efficiency caused by structural parameters of the secondary wall thickening vessel. Among the vessels with four cross-sectional types, the circular vessel had the highest water transmission efficiency, and the quadrilateral vessel had the lowest water transmission efficiency, indicating that the polygon vessel with fewer sides had a lower water transmission efficiency. This also explained that the vessel used hexagonal and circular cross-section as the main water transport channel.

The increasing and decreasing trend of the structure coefficient and the flow resistance coefficient were consistent with the variation of annular (pitted) width, annular (pitted) height, and annular (pitted) spacing, and opposite in the variation of annular (pitted) inscribed circle diameter. The annular and pitted thickening vessels with larger inscribed circle diameters had lower flow resistance and lower water transport efficiency, which demonstrated that secondary wall thickening structure restricted the inner diameter of vessel, because the vessel structure should maintain the balance between flow resistance and transport efficiency [7]. The longer and wider single vessel, the greater the proportion of transport capacity that will be lost if the vessel becomes embolized [25, 26]. This was shown that the water transfer efficiency of the annular thickening and the pitted thickening vessel was different (Figs 9 and 10). The structure coefficient of the pitted thickening vessel was significantly lower than that of annular thickening vessel in hexagon vessel. The reason was that pitted thickening vessel was close to the ideal smooth vessel, which also explained that the secondary thickening vessel of plants was the evolution of annular thickening to pitted thickening [18, 21].

## Conclusion

There was annular thickening and pitted thickening walls in *Panax notoginseng* xylem vessels. The flow resistance of the annular thickening vessel was significantly higher than that of pitted thickening vessel in four cross-sectional types. The influence degree of cross-sectional type on the flow resistance was clarified in descending order: circular, hexagon, pentagon and quadrilateral section.

Through the simulations and related calculations of vessel model, a large amount of the data between the *ξ* and structural parameters was obtained. The *ξ* was positively correlated with the annular height, pitted width and pitted height, and negatively correlated with the annular inscribed circle diameter, annular width, annular spacing, pitted inscribed circle diameter and pitted spacing. Among them, annular (pitted) height and the annular (pitted) inscribed circle diameter had a great influence on the *ξ*.

This paper studied the relationship between the *S* and *ξ*, it was found that the increasing and decreasing trend of two parameters were opposite in the change of annular (pitted) inscribed circle diameter, and consistent in the change of in other structural parameters, indicating that the secondary wall thickening structure limited the inner diameter of the vessel to maintain a balance between flow resistance and transport efficiency.

At present, the research focuses on the influence of xylem vessel structure on water transport. The future work could consider the influence of environment on the growth of plant xylem and expound the multiple trade-off relationship among environment-xylem structure-efficiency.

## Supporting information

S1 Table The annular thickening vessel parameters on flow resistance coefficient. (DOCX)

S2 Table The pitted thickening vessel parameters on flow resistance coefficient. (DOCX)
