# Supplementary material for: Xylem vessel type and structure influence the water transport characteristics of Panax notoginseng
Source: PLoS One. 2023 Mar 6;18(3):e0281080. doi: 10.1371/journal.pone.0281080 (PMC9987790; doi:10.1371/journal.pone.0281080)
Supplement: S1 Table — (DOCX) [file pone.0281080.s001.docx]

**Table 1. The annular thickening vessel parameters on flow resistance coefficient**

| Parameters (µm) | Annular thickening | | |
| --- | --- | --- | --- |
|  | Δp/Pa | q/(m^3^·s^–1^) | *ξ* |
| Annular inscribed circle diameter (18µm) | 20.86 | 8.40×10^-14^ | 6.09×10^5^ |
| Annular inscribed circle diameter (20µm) | 15.19 | 1.04×10^-13^ | 2.73×10^5^ |
| Annular inscribed circle diameter (22µm) | 11.57 | 1.26×10^-13^ | 1.31×10^5^ |
| Annular inscribed circle diameter (24µm) | 9.13 | 1.49×10^-13^ | 0.67×10^5^ |
| Annular width (2µm) | 15.49 | 1.04×10^-13^ | 2.80×10^5^ |
| Annular width (2.2µm) | 15.31 | 1.04×10^-13^ | 2.76×10^5^ |
| Annular width (2.4µm) | 15.19 | 1.04×10^-13^ | 2.73×10^5^ |
| Annular width (2.6µm) | 15.09 | 1.04×10^-13^ | 2.71×10^5^ |
| Annular height (2µm) | 12.71 | 1.04×10^-13^ | 2.18×10^5^ |
| Annular height (2.2µm) | 13.88 | 1.04×10^-13^ | 2.44×10^5^ |
| Annular height (2.4µm) | 15.19 | 1.04×10^-13^ | 2.73×10^5^ |
| Annular height (2.6µm) | 16.71 | 1.04×10^-13^ | 3.07×10^5^ |
| Annular spacing (2µm) | 15.47 | 1.04×10^-13^ | 2.79×10^5^ |
| Annular spacing (2.2µm) | 15.27 | 1.04×10^-13^ | 2.75×10^5^ |
| Annular spacing (2.4µm) | 15.19 | 1.04×10^-13^ | 2.73×10^5^ |
| Annular spacing (2.6µm) | 15.10 | 1.04×10^-13^ | 2.71×10^5^ |
