# Supplementary material for: Xylem vessel type and structure influence the water transport characteristics of Panax notoginseng
Source: PLoS One. 2023 Mar 6;18(3):e0281080. doi: 10.1371/journal.pone.0281080 (PMC9987790; doi:10.1371/journal.pone.0281080)
Supplement: S2 Table — (DOCX) [file pone.0281080.s002.docx]

**Table 2. The pitted thickening vessel parameters on flow resistance coefficient**

| Parameters (µm) | Pitted thickening | | |
| --- | --- | --- | --- |
|  | Δp/Pa | q/(m^3^·s^–1^) | *ξ* |
| Pitted inscribed circle diameter (18µm) | 7.92 | 8.40×10^-14^ | 16.91×10^4^ |
| Pitted inscribed circle diameter (20µm) | 6.52 | 1.04×10^-13^ | 7.99×10^4^ |
| Pitted inscribed circle diameter (22µm) | 5.44 | 1.26×10^-13^ | 3.80×10^4^ |
| Pitted inscribed circle diameter (24µm) | 4.62 | 1.49×10^-13^ | 1.80×10^4^ |
| Pitted width (2µm) | 6.53 | 1.04×10^-13^ | 8.00×10^4^ |
| Pitted width (3µm) | 6.57 | 1.04×10^-13^ | 8.10×10^4^ |
| Pitted width (4µm) | 6.60 | 1.04×10^-13^ | 8.15×10^4^ |
| Pitted width (5µm) | 6.63 | 1.04×10^-13^ | 8.23×10^4^ |
| Pitted height (1µm) | 6.29 | 1.08×10^-13^ | 6.89×10^4^ |
| Pitted height (1.2µm) | 6.53 | 1.04×10^-13^ | 8.00×10^4^ |
| Pitted height (1.4µm) | 6.78 | 9.96×10^-14^ | 9.29×10^4^ |
| Pitted height (1.6µm) | 7.04 | 9.56×10^-14^ | 10.76×10^4^ |
| Pitted spacing (2µm) | 6.69 | 1.04×10^-13^ | 8.36×10^4^ |
| Pitted spacing (3µm) | 6.60 | 1.04×10^-13^ | 8.16×10^4^ |
| Pitted spacing (4µm) | 6.53 | 1.04×10^-13^ | 8.00×10^4^ |
| Pitted spacing (5µm) | 6.45 | 1.04×10^-13^ | 7.81×10^4^ |
